# ADPCNet: Adaptive Deformable Peripheral Convolution for Efficient Image Dehazing

**DOI:** 10.3390/jimaging12060235

**Published:** 2026-05-28

**Authors:** Zhihao Wang, Yunjie Zhu, Xiaolong Zheng, Suyu Yang, Chunhua Hu

**Affiliations:** College of Information Science and Technology & College of Artificial Intelligence, Nanjing Forestry University, Nanjing 210037, China; zhwang@njfu.edu.cn (Z.W.); aylward@njfu.edu.cn (Y.Z.); zhengxiaolong@njfu.edu.cn (X.Z.); 8250811202@njfu.edu.cn (S.Y.)

**Keywords:** image dehazing, image restoration, large-kernel convolution, peripheral convolution, deformable sampling, frequency-guided modulation, efficient neural networks

## Abstract

Single-image dehazing requires wide-range visibility estimation and local structure recovery under spatially varying degradation. Existing large-context models improve global reasoning, but they often incur heavy computation or lose sensitivity to irregular haze boundaries and attenuated details. To address these issues, we propose the Adaptive Deformable Peripheral Convolution Network (ADPCNet), a compact encoder–decoder that organizes dehazing into four coupled operations: conditional adaptive sharing for peripheral large-kernel context modeling, deformable sampling for geometry-aware aggregation, frequency-guided modulation for detail compensation, and dynamic multi-branch fusion for content-adaptive integration. The key idea is to separate broad haze estimation, structure alignment, and detail recovery within an efficient operator stack. Experiments on RESIDE, Dense-Haze, and NH-Haze show that ADPCNet achieves competitive paired-benchmark performance with 7.25 M parameters and 33.62 G FLOPs, reaching 40.89 dB/0.997 on SOTS-Indoor, 37.80 dB/0.996 on SOTS-Outdoor, 18.05 dB/0.679 on Dense-Haze, and 21.66 dB/0.815 on NH-Haze. The ablation and sensitivity results further support the contributions of the proposed modules and the selected kernel configuration. Overall, these results indicate that ADPCNet maintains a favorable quality-efficiency trade-off under the matched paired evaluation protocol.

## 1. Introduction

Single-image dehazing is a representative inverse imaging problem in which atmospheric scattering simultaneously reduces contrast, suppresses high-frequency details, and distorts scene colors. The degradation is especially severe in outdoor vision systems, where haze directly affects traffic monitoring, autonomous navigation, remote inspection, and adverse-weather perception. Unlike blur or additive noise, haze is strongly depth-coupled and spatially non-uniform. The same image may contain weak haze in nearby regions, dense haze in distant regions, and abrupt transitions around object boundaries. Effective dehazing therefore requires broad contextual reasoning together with precise local restoration. The dark channel prior is one of the most influential physical priors for single-image haze removal [1], and standard paired benchmarks, especially RESIDE and its SOTS subsets, have shaped the quantitative evaluation protocol of supervised single-image dehazing [2].

Classical dehazing methods are derived from the atmospheric scattering model and rely on handcrafted priors. Early visibility restoration and statistical dehazing methods established important formulations for single-image bad-weather restoration [3,4,5]. The color attenuation prior, edge-preserving decomposition, haze-line modeling, and air-light estimation further extended handcrafted dehazing priors from different image statistics [6,7,8,9]. The dark channel prior was also generalized for broader restoration settings [10]. These methods established the physical foundation of the field, but their assumptions are easily violated by sky regions, bright objects, artificial lighting, and non-homogeneous haze. Once the prior fails, residual haze, halo artifacts, and color distortion are difficult to avoid.

Deep learning changed the landscape by replacing explicit transmission estimation with direct image restoration. DehazeNet and MSCNN introduced early CNN-based transmission estimation and multi-scale prediction [11,12]. AOD-Net and DCPDN further connected neural restoration with the atmospheric scattering model through reformulation or joint physical-prior learning [13,14]. Later CNN-based models improved gated context aggregation, grid-based multi-scale interaction, attention fusion, and boosted dense restoration [15,16,17,18,19]. Physical-prior-guided, multiscale attention, and edge-aware variants further improved robustness and structural preservation [20,21,22]. Transformer and hybrid models then expanded global modeling through transmission-aware position encoding, density-aware restoration, guided fusion, DehazeFormer-style design, mixed structural blocks, and diffusion priors [23,24,25,26,27,28,29,30]. These advances clearly show that dehazing benefits from long-range interaction and stronger feature modulation; however, they also reveal sharper trade-offs among restoration quality, model size, memory footprint, and deployment cost.

Large-kernel convolution has emerged as an effective alternative to heavy self-attention in low-level vision. Recent restoration studies show that carefully designed large-kernel operators can capture long-range dependencies while preserving the optimization stability and deployment simplicity of convolutional networks. For dehazing, this direction is particularly attractive because haze statistics are globally correlated: distant haze thickness, scene illumination, and color shift are rarely determined by a tiny local neighborhood. At the same time, directly enlarging the kernel is not sufficient. Dense large kernels increase parameters and FLOPs rapidly, fixed-grid aggregation is poorly matched to irregular haze boundaries, and broad context aggregation often suppresses the high-frequency cues that determine visually convincing restoration.

These observations reveal a specific operator mismatch in dehazing. Broad context is needed to estimate slowly varying atmospheric veiling, but that context must be aligned to irregular scene geometry, and the resulting response must still recover high-frequency details that haze suppresses most strongly. Practical architecture therefore needs three properties at the same time: efficient large-range context coverage, structure-aware sampling, and explicit detail compensation. Existing methods usually focus on only one or two of these properties, often leading to issues such as incomplete dense haze removal, misaligned scene structures, loss of high-frequency details, or amplified noise, making it difficult to achieve robust and clear dehazing effects under a lightweight architecture.

To address this gap, we propose the Adaptive Deformable Peripheral Convolution Network (ADPCNet). CASM first establishes a parameter-efficient peripheral large kernel for broad haze modeling. DSM then relocates the effective support of that kernel near boundaries and thin structures, preventing fixed-grid aggregation from mixing incompatible regions. FGM restores attenuated edges and texture transitions after context aggregation, and DMF finally balances local, macro, and spectral cues according to image content. The four are organized as modules of successive responses to the same dehazing contradiction: broad-range estimation is necessary, yet it is only effective when followed by geometry-aware support correction and detail compensation.

The main contributions of this work are summarized as follows.

1. We propose ADPCNet, a dehazing-oriented operator stack that couples peripheral large-kernel context modeling, deformable alignment, frequency-guided detail compensation, and dynamic branch fusion in a unified encoder–decoder framework.
2. We formulate CASM as a content-adaptive large-kernel operator that reduces peripheral redundancy while preserving long-range contextual aggregation.
3. We introduce DSM and FGM to address two persistent weaknesses of large-context restoration, namely structural misalignment and detail attenuation.
4. We design DMF for adaptive fusion of context, structure, and detail branches.
5. We evaluate ADPCNet on standard paired benchmarks covering synthetic and real hazy scenes and provide unified parameter/FLOP comparisons together with ablation analysis.

## 2. Related Work

### 2.1. Prior-Based Single-Image Dehazing

Prior-based dehazing methods explicitly model atmospheric scattering and estimate transmission of air-light by exploiting the statistical regularities of natural images. The dark channel prior is one of the most influential physical priors for single-image haze removal [1]. Early visibility restoration and statistical dehazing methods established important formulations for single-image bad-weather restoration [3,4,5]. The color attenuation prior, edge-preserving decomposition, haze-line modeling, and air-light estimation further extended handcrafted dehazing priors from different image statistics [6,7,8,9]. The dark channel prior was also generalized for broader restoration settings [10]. These methods remain conceptually important because they make the depth-dependent nature of haze explicit. Their limitation is not the physical model itself, but the rigidity of handcrafted assumptions. In scenes containing bright objects, dense sky regions, or strongly non-homogeneous haze, the estimated transmission often becomes unstable, which directly harms the final restoration quality.

### 2.2. CNN-Based Dehazing Networks

CNN-based dehazing networks established the dominant supervised paradigm for the field. DehazeNet and MSCNN introduced learnable transmission estimation and multi-scale convolutional prediction [11,12]. AOD-Net directly generated the clean image through a reformulated scattering model, while DCPDN combined physical priors with densely connected restoration [13,14]. GCA-Net and GridDehazeNet strengthened gated context aggregation and attention-based multi-scale interaction [15,16]. Enhanced Pix2Pix dehazing and FFA-Net further explored image-to-image translation and feature-fusion attention [17,18]. MSBDN, PSD, MSAFF-Net, and edge-aware variants improved dense multi-scale fusion, synthetic-to-real transfer, multiscale attention, and boundary-sensitive restoration [19,20,21,22]. Their main strengths are stable optimization and efficient inference. Their main weakness is receptive-field efficiency: when broader context is required, the network usually must grow deeper, wider, or more densely connected. ADPCNet stays within the convolutional regime but replaces naive depth expansion with a more structured large-kernel design.

### 2.3. Transformer and Hybrid Dehazing Models

Transformer-based and hybrid dehazing models further improved global modeling by introducing attention-guided feature mixing. DeHamer and density-aware dehazing models introduced transmission-aware positional modeling and haze-density perception [23,24]. MSGC-Net and self-guided progressive fusion improved context aggregation and feature refinement [25,26]. Dehazing performance analysis and DehazeFormer further clarified the role of architecture design in Transformer-based dehazing [27,28]. MixDehazeNet and diffusion-based dehazing expanded this line toward mixed structural blocks and generative restoration priors [29,30]. These methods demonstrate that long-range dependency modeling is beneficial for dehazing, especially in scenes with large smooth regions and complex depth layout. However, full attention is not always the most efficient way to obtain global context when inference cost and memory usage are part of the design target.

### 2.4. Recent Strong Baselines and Large-Kernel Restoration

The line most directly related to ADPCNet is the recent shift from generic depth expansion to efficient global-context operators. FGN and LKD-Net improve guidance and large-kernel dehazing [31,32]. DehazeDCT and CasDyF-Net introduce deformable Transformer modeling and cascaded dynamic filtering for non-homogeneous or adaptive restoration [33,34]. DRACO-DehazeNet and RIDCP further emphasize detail recovery, contrastive learning, and high-quality real-image priors [35,36]. IPC-Dehaze, Diff-Dehazer, and realistic haze-generation methods address real-world or unpaired dehazing from code decoding, diffusion priors, or degradation modeling perspectives [37,38,39]. Frequency-domain diffusion, remote-sensing dehazing, and elementary function fusion further expand the recent dehazing design space [40,41,42]. These developments are also connected to the broader Transformer and vision-backbone literature, including self-attention, Vision Transformer, and Swin Transformer [43,44,45]. In parallel, OKNet and ConvIR revisit efficient convolutional restoration with strong global-to-local representation capacity [46,47]. FSNet and SANet introduce frequency selection and strip attention for restoration [48,49]. MAXIM and Instruct-IPT represent MLP/Transformer-style all-in-one restoration backbones [50,51]. PELK, RepLKNet, and SLaK further demonstrate the potential of parameter-efficient or sparse large-kernel convolution [52,53,54]. The unresolved issue is not whether broad context helps, but how to realize it in a dehazing-specific operator that also handles structural misalignment and detail attenuation without sacrificing efficiency.

### 2.5. Deformable and Frequency-Aware Modeling

Deformable convolution and frequency-aware modulation address two weaknesses that remain after broad context is introduced. Deformable convolution introduces learnable offsets so that feature sampling can adapt to object geometry and local structures [55]. Deformable ConvNets v2 further improves deformable sampling through stronger modulation and refinement [56]. Frequency-aware restoration is equally relevant because haze suppresses edge contrast and fine textures more strongly than low-frequency content [48]. ADPCNet integrates these corrections after large-context modeling, so that contextual estimation, structural alignment, and detail recovery are handled as distinct but connected stages. Accordingly, ADPCNet is positioned as a compact dehazing-oriented operator stack that jointly considers large-context visibility estimation, geometry-aware support correction, and frequency-sensitive detail recovery.

## 3. Materials and Methods

### 3.1. Problem Formulation

Given a hazy input image $I\in R^{H\times W\times 3}$, the goal of single-image dehazing is to recover the corresponding haze-free image $J\in R^{H\times W\times 3}$. The atmospheric scattering model is commonly written as

(1) $$I\left(x\right)=J\left(x\right)t\left(x\right)+A(1-t(x)),$$

where x denotes a spatial location, $t\left(x\right)$ is the transmission map, and A is the global atmospheric light. Although this model offers useful physical intuition, the present work does not explicitly estimate $t\left(x\right)$ or A. Instead, we learn an end-to-end mapping

(2) $$\hat{J}=F_{\theta }(I),$$

where $F_{\theta }$ is parameterized by ADPCNet. This choice avoids repeated hand-designed estimation steps and allows the model to directly optimize image restoration quality. Nevertheless, the network design remains guided by the degradation properties implied by atmospheric scattering. ADPCNet does not explicitly estimate physical variables such as the transmission map or atmospheric light. Instead, it organizes the learned features into several functional representations: broad haze-context representation for spatially varying visibility estimation, geometry-aware sampling representation for local structural alignment, frequency-sensitive representation for attenuated detail compensation, and spatial confidence representation for adaptive evidence fusion. This interpretation links the physical motivation of haze degradation with the internal feature organization of the proposed network.

### 3.2. Overall Architecture

**Figure 1:**
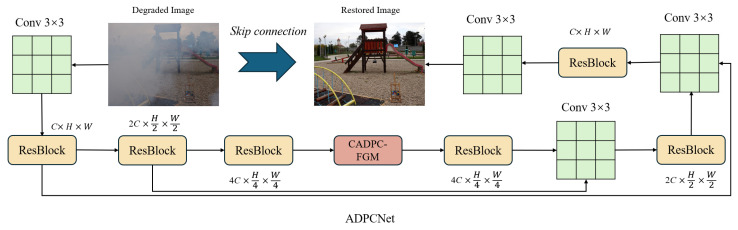
Overall architecture of ADPCNet. The network adopts a hierarchical encoder–bottleneck–decoder design with shallow embedding, multi-stage restoration blocks, skip connections, and residual reconstruction.

ADPCNet follows a hierarchical encoder–bottleneck–decoder architecture with shallow embedding, multi-stage feature transformation, and residual image reconstruction. Given the input image I, a 3×3 embedding convolution first projects the RGB image into a feature space $F_{0}$. The overall architecture of ADPCNet is shown in Figure 1. The encoder then produces stage-wise features

(3) $$F_{s}=E_{s}(F_{s-1}),s=1,2,\ldots ,S,$$

where each encoder stage $E_{s}(\cdot )$ contains the proposed restoration block and a downsampling operator. The bottleneck aggregates the deepest contextual representation

(4) $$F_{b}=B(F_{S}),$$

and the decoder reconstructs the clean image through progressive up-sampling and skip fusion,

(5) $$tildeF_{s-1}=D_{s-1}(F_{s}^{↑},F_{s-1}),$$

(6) $$\hat{J}=I+R({\tilde{F}}_{0}).$$

where $F_{s}^{↑}$ denotes the upsampled decoder feature and $R({\tilde{F}}_{0})$ is the reconstruction head. The residual formulation stabilizes optimization and allows the network to focus on haze-induced distortion.

Each restoration block contains three explicit feature branches followed by adaptive fusion. Let F denote the block input. The local branch preserves short-range structures by applying the independent central kernel region and produces $F_{loc}$. The macro branch first forms a conditionally adapted 63×63 peripheral kernel through CASM and then applies DSM to generate a geometry-aware large-kernel response $F_{mac}$. In parallel, the spectral branch transforms F into the frequency domain, predicts a frequency attention map, and returns the enhanced feature $F_{spec}$. DMF then fuses the three branches as

(7) $$F_{\mathrm{blk}}=F_{\mathrm{DMF}}(F_{\mathrm{loc}},F_{\mathrm{mac}},F_{\mathrm{spec}}),$$

the fused feature is then propagated to the next encoder or decoder stage. Under this branch-wise interpretation, ADPCNet decomposes the dehazing process into four connected representation roles: local spatial evidence preservation, large-range haze-context estimation, geometry-aware support correction, and frequency-sensitive detail compensation. The fusion weights learned by DMF further act as spatial confidence maps that determine which representation is more reliable at each location.

The ordering of these components follows their functional dependencies. CASM must precede DSM because offset learning is only meaningful after a wide-support kernel has been defined. DSM must operate before fusion because the macro response should be aligned to scene structure before it competes with local and spectral cues. FGM is kept as a parallel correction branch so that detail-sensitive responses are recovered without being repeatedly diffused by large-context aggregation. DMF then reconciles the three cue types spatially. In this way, each block resolves one dehazing contradiction step by step: estimate broad haze tendency, correct structural support, recover attenuated detail, and fuse the resulting evidence according to local content.

### 3.3. Conditional Adaptive Sharing Module

The purpose of CASM is to retain the contextual benefit of large-kernel convolution without paying the full cost of a dense kernel. This design is related to efficient convolutional restoration and parameter-efficient or sparse large-kernel modeling [46,47,52,53,54]. ADPCNet adopts a fixed peripheral-convolution layout in which the central region keeps independent parameters and the outer area is generated from a shared trainable kernel through the peripheral sharing grid. This design preserves fine-grained local modeling near the kernel center while compressing the parameterization of long-range context. In the implemented configuration, the peripheral support is set to 63 × 63, and the central 7 × 7 region keeps independent parameters for local detail modeling.

Let $F\in R^{H\times W\times C}$ denote an intermediate feature map. A lightweight auxiliary predictor generates a spatial gate map g(p)∈[0,1] for each position p. The position-dependent peripheral kernel is written as

(8) $$W_{\mathrm{casm}}(p)=g(p)⊙W_{\mathrm{shr}}(p)+(1-g(p))⊙W_{\mathrm{ind}}(p),$$

where $W_{\mathrm{shr}}$ denotes the shared peripheral kernel weights and $W_{\mathrm{ind}}$ denotes the independent kernel weights retained for detail-critical responses. The gate is spatially defined, allowing each location to decide how strongly the block relies on shared peripheral parameters.

The corresponding CASM output is formed by combining the fixed central kernel response with the gated peripheral response,

(9) $$F_{\mathrm{casm}}(p_{0})=\sum_{q\in R_{7}}W_{\mathrm{cen}}(q)\cdot F(p_{0}+q)+\sum_{q\in R_{63}\setminus R_{7}}W_{\mathrm{casm}}(q;p_{0})\cdot F(p_{0}+q),$$

where $R_{7}$ denotes the central 7×7 support and $R_{63}\backslash R_{7}$ denotes the peripheral region. In dehazing terms, smooth regions can exploit strong peripheral sharing, whereas detail-rich or boundary-heavy regions preserve more position-specific weights. CASM therefore expands the receptive field while keeping parameter growth moderate and provides the large-context basis used by the macro branch. In terms of internal representation, the CASM response is interpreted as a broad haze-context feature. The spatial gate indicates how strongly each location should rely on shared peripheral context, which is useful for modeling slowly varying haze distribution while retaining position-specific responses in detail-rich or boundary-heavy regions.

### 3.4. Deformable Sampling Module

Large receptive fields improve contextual reasoning, but they also increase the risk of mixing incompatible regions across depth boundaries. This issue is especially evident near vegetation, traffic signs, wires, and building contours, where haze thickness changes abruptly. DSM addresses this problem by replacing rigid lattice sampling with content-adaptive sampling. Deformable convolution introduced learnable sampling offsets for adaptive spatial support [55], and Deformable ConvNets v2 further improved deformable sampling and modulation [56].

DSM operates on the macro branch after CASM has produced the conditionally adapted large-kernel weights. Given an input feature map F, an offset head predicts sampling offsets $\Delta p_{n}(p_{0})$ for each reference point $p_{n}\in R_{63}$ of the fixed 63×63 sampling grid. The macro-branch output at location $p_{0}$ is

(10) $$F_{\mathrm{mac}}(p_{0})=\sum_{p_{n}\in R_{63}}W_{\mathrm{casm}}(p_{n};p_{0})\cdot F(p_{0}+p_{n}+\Delta p_{n}(p_{0})),$$

where bilinear interpolation is used for fractional coordinates. The learned offsets represent adaptive sampling support inferred from residual structural evidence in the learned feature space. When weak contours, depth transitions, or thin structures remain observable, DSM shifts the effective sampling positions toward geometry-relevant locations, thereby reducing the risk of mixing incompatible regions within a large receptive field.

This coupling is structurally important. CASM provides the large-context kernel, while DSM determines where that kernel should sample. The macro branch therefore combines long-range haze modeling with geometry-aware aggregation instead of applying a fixed large support everywhere.

### 3.5. Frequency-Guided Modulation

Haze suppresses high-frequency content more strongly than low-frequency content, so a purely context-dominant network tends to recover visibility first and detail later. FGM is introduced to correct this imbalance by explicitly modulating the spectrum of the intermediate feature. Frequency selection is relevant because restoration quality often depends on frequency-sensitive feature modulation [48].

Let F be the incoming feature map. FGM first computes the frequency-domain representation

(11) $$\hat{F}=FFT(F),$$

where $FFT\left(\cdot \right)$ denotes the Fast Fourier Transform. Then, a lightweight convolutional predictor generates a frequency attention map $A_{\mathrm{freq}}$, and the spectral branch output is obtained as

(12) $$F_{\mathrm{spec}}=IFFT(\hat{F}⊙A_{\mathrm{freq}}),$$

where $IFFT\left(\cdot \right)$ denotes the inverse Fast Fourier Transform. The branch is thus defined by explicit FFT-domain attention. The inverse-transformed feature keeps the same spatial size and channel layout as the input feature, so it can be fused directly with the local and macro branches without extra resampling or stage-specific adaptation.

FGM complements the spatial branches by providing a frequency-sensitive detail representation. It strengthens recoverable high-frequency responses that remain weak after large-context aggregation. In this sense, the spectral feature $F_{\mathrm{spec}}$ represents detail-compensation evidence that is later balanced with local spatial evidence and macro contextual responses through DMF.

### 3.6. Dynamic Multi-Branch Fusion

The local, macro, and spectral branches provide complementary restoration cues. Context-heavy responses are more useful in dense haze or smooth sky regions, while structure-heavy and detail-heavy responses become more important near boundaries and thin textures. DMF learns this spatially varying balance explicitly.

After channel alignment, the three branch features $\left\{F_{\mathrm{loc}},F_{\mathrm{mac}},F_{\mathrm{spec}}\right\}$ are fed to a dynamic gating network that predicts three spatial weight maps. The normalized weight of branch i at position p is

(13) $${\hat{\alpha }}_{i}(p)=\frac{\exp\left(\gamma_{i}A_{i}(p)\right)}{\sum_{j\in \{\mathrm{loc},\mathrm{mac},\mathrm{spec}\}}exp(\gamma_{j}A_{j}(p))},$$

$A_{i}(p)i\gamma_{i}$ where $A_{i}(p)$ is the predicted gating response of branch i and $\gamma_{i}$ is its scale factor. The fused output is then

(14) $$F_{\mathrm{out}}(p)={\hat{\alpha }}_{\mathrm{loc}}(p)⊙F_{\mathrm{loc}}(p)+{\hat{\alpha }}_{\mathrm{mac}}(p)⊙F_{\mathrm{mac}}(p)+{\hat{\alpha }}_{\mathrm{spec}}(p)⊙F_{\mathrm{spec}}(p).$$

DMF is therefore implemented as a softmax-normalized spatial fusion layer. Because the three branch outputs are aligned before gating, the fusion stage only needs to predict spatial confidence maps and does not introduce additional cross-scale reconstruction logic.

**Figure 2:**
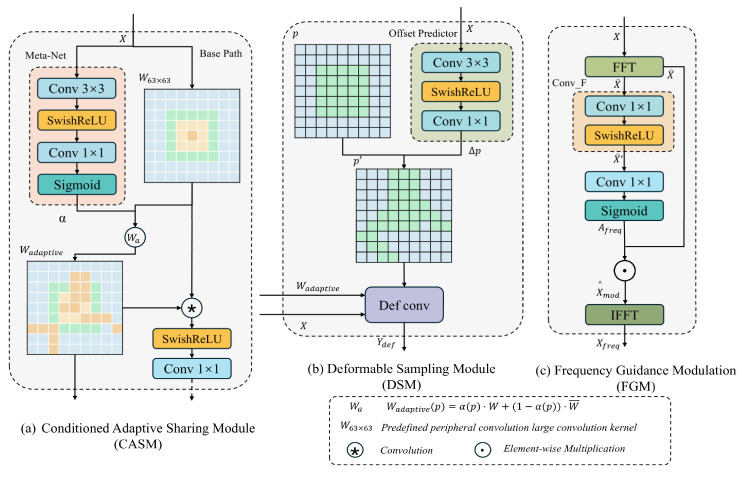
Detailed structures of CASM, DSM, and FGM. (**a**): CASM generates a content-adaptive peripheral large-kernel response through a meta-network and adaptive kernel sharing. (**b**): DSM predicts spatial offsets and performs deformable convolution to align the sampling support with geometry-relevant structures. (**c**): FGM applies FFT-based frequency modulation and IFFT reconstruction to enhance recoverable high-frequency responses. Different colors indicate different functional components, and arrows indicate feature propagation directions.

DMF is important for stability as well as accuracy. Without adaptive fusion, the strongest branch tends to dominate the response, which can either over-smooth the output or over-enhance local textures. DMF suppresses such uniform behavior by allowing the model to emphasize the most reliable branch for each region. The predicted fusion weights can therefore be viewed as spatial confidence maps over the local, macro, and spectral representations. In dense haze or smooth regions, the macro contextual response may receive higher confidence, whereas near boundaries or texture-rich areas, the local and spectral responses may become more important. This design makes the final restoration feature interpretable as a content-adaptive integration of context, structure, and detail evidence. The detailed structures of CASM, DSM, and FGM are illustrated in Figure 2.

CASM constructs parameter-efficient peripheral large-kernel context, DSM relocates the effective sampling support to geometry-relevant positions, and FGM restores attenuated high-frequency responses through frequency-guided modulation.

**Figure 3:**
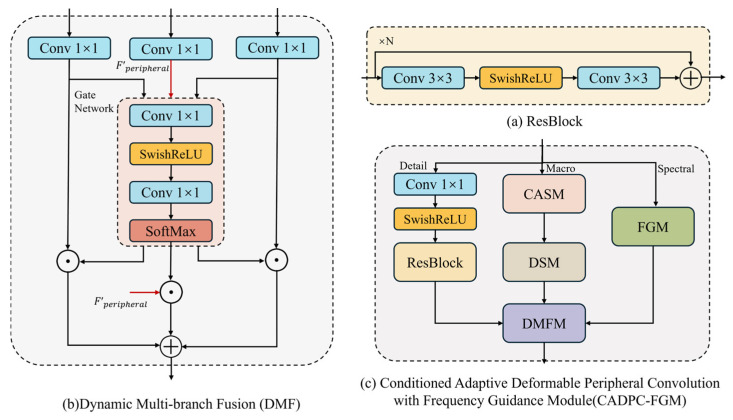
Detailed structure of DMFM, ResBlock, and the restoration block. (**a**): DMFM performs spatially adaptive branch fusion through gate prediction and element-wise modulation. (**b**): ResBlock contains two 3 × 3 convolutional layers with SwishReLU activation and a residual connection. (**c**): The restoration block organizes detail, macro, and spectral branches and fuses them through DMFM. Different colors indicate functional components, arrows indicate feature propagation directions, the circled-dot symbol denotes element-wise modulation or gating, and the circled-plus symbol denotes feature addition. DMF adaptively fuses the local, macro, and spectral branches through spatially normalized gating, allowing the network to balance context estimation, structure-aware aggregation, and detail refinement according to image content.

The DMF structure and branch interaction are shown in Figure 3.

### 3.7. Detail Restoration and Reconstruction Path

Skip connections transfer shallow spatial information directly to the decoder, which is essential for recovering edges and thin textures that weaken after repeated downsampling. Let $F_{s}$ denote the encoder feature at stage s and ${\hat{F}}_{s}$ denote the decoder feature at the same scale. The skip-fusion process is written as

(15) $${\hat{F}}_{s}=U_{s}({\hat{F}}_{s+1})+S_{s}(F_{s}),$$

where $U_{s}(\cdot )$ denotes upsampling and $S_{s}(\cdot )$ denotes a skip projection for channel alignment. The final reconstruction head is a convolutional residual predictor applied to ${\hat{F}}_{0}$, and the restored image is recovered as $\hat{J}=I+R({\hat{F}}_{0})$. This design concentrates the network capacity on haze removal, contrast recovery, and detail refinement.

The detail path is particularly important after DMF. Once context, structure, and frequency cues have been fused, the reconstruction head converts this fused representation into visually consistent image content. The ablation study confirms that this detail-oriented reconstruction path contributes measurable gains at low additional cost.

In ADPCNet, the detail branch is not an additional branch after fusion; it is the local branch that enters DMF together with the macro and spectral branches. The reconstruction head therefore receives a decoder feature that has already integrated local detail, macro context, and spectral correction.

### 3.8. Optimization Objective

Under the dehazing setting, ADPCNet is optimized end to end on hazy/clear image pairs $\{(I_{n},J_{n}){\}}_{n=1}^{N}$. The training objective is defined at the restored-image level as

(16) $$\theta^{*}=arg\;\underset{\theta }{min}\frac{1}{N}\sum_{n=1}^{N}D(F_{\theta }(I_{n}),J_{n}),$$

where $D(F_{\theta }(I_{n}),J_{n})$ denotes the supervised reconstruction objective between the restored image and the ground-truth clear image. The objective is applied directly to the final restored RGB output. The staged training procedure described in Section 4.2 initializes the auxiliary predictors in CASM, DSM, and FGM before switching to joint optimization under this image-level objective. In practice, gradients from D jointly optimize the restoration backbone, sharing gates, sampling offsets, spectral attention, and fusion weights, without introducing separate branch-specific losses.

### 3.9. Complexity Considerations

Efficiency is quantified under the common 256×256 input protocol used in the main comparison table. Under this setting, ADPCNet contains 7.25 M parameters and 33.62 G FLOPs, compared with 14.42 M parameters and 41.86 G FLOPs for OKNet. This corresponds to a parameter reduction of approximately 49.7% and a FLOP reduction of approximately 19.7% relative to OKNet under the same table protocol.

All FLOP statistics were recalculated under the same 256 × 256 input protocol and the same counting script. The reported full-model FLOPs include the restoration backbone, auxiliary predictors in CASM/DSM/FGM, dynamic fusion, and reconstruction head. Accordingly, 33.62 G is used as the unified full-inference FLOP value for the default ADPCNet configuration throughout the manuscript.

## 4. Experimental Setup

### 4.1. Datasets

ADPCNet is evaluated on both synthetic and real-world dehazing benchmarks. The synthetic evaluation follows the RESIDE benchmark family, with SOTS-Indoor and SOTS-Outdoor used for cross-method quantitative comparison [2]. The main training and ablation protocol is built around the outdoor setting, which is consistent with the original ADPCNet comparison and with the 30-epoch RESIDE-Outdoor ablation setting described in the experimental section. SOTS-Indoor is retained because it remains a standard reference benchmark and is reported by nearly all major competing methods.

Real-world evaluation is conducted on Dense-Haze and NH-Haze, using their original paired benchmark definitions [57,58]. Dense-Haze emphasizes severe visibility degradation and tests whether the model can recover global contrast without introducing color distortion [57]. NH-Haze contains spatially varying haze patterns and is more sensitive to local structural errors [58]. O-HAZE and recent NTIRE dense and non-homogeneous dehazing challenges further show the continued importance of real paired and challenge-style dehazing evaluation [59,60]. These datasets are therefore well aligned with the design of DSM and DMF, which explicitly target non-uniform haze and geometry-aware feature aggregation.

### 4.2. Implementation Details

ADPCNet was implemented using PyTorch 1.8.1 under Python 3.8, CUDA 10.2, and cuDNN 7.6.5. All experiments were conducted on an Ubuntu server equipped with an NVIDIA GeForce RTX 4090 GPU (NVIDIA Corporation, Santa Clara, CA, USA). Training used fully preprocessed paired images. AdamW was adopted as the optimizer with $\beta_{1}=0.9$, $\beta_{2}=0.999$, and weight decay equal to 0.05. The batch size was set to 8. To improve robustness and generalization, the training pipeline used RandAugment, Mixup with coefficient 0.8, and CutMix with coefficient 1.0. Because CASM, DSM, and FGM contain auxiliary prediction subnetworks, the training schedule first initializes these predictors separately and then switches to gradual joint optimization of the full network. For ablation, the model is trained for 30 epochs on RESIDE-Outdoor and evaluated on SOTS-Outdoor. All cross-method complexity statistics follow the unified 256 × 256 input protocol described above.

### 4.3. Compared Methods

We compare ADPCNet with representative prior-based, CNN-based, transformer-based, large-kernel, and diffusion-related dehazing methods. The main quantitative comparison is organized around the paired benchmark setting, where SOTS-Indoor, SOTS-Outdoor, Dense-Haze, and NH-Haze are used as the primary full-reference evaluation datasets. Here, “paired” means that each hazy image has a corresponding haze-free reference image, which enables full-reference evaluation using PSNR and SSIM. To avoid overstating cross-protocol comparisons, direct quantitative claims are made only for methods whose reported results overlap with this paired benchmark setting. Recent methods evaluated under challenge-style, unpaired, no-reference, or real-world-only protocols are discussed as related context. Together with the ablation study and complexity analysis, this comparison protocol is used to evaluate both restoration performance and model efficiency.

### 4.4. Evaluation Metrics

For paired benchmarks, PSNR and SSIM are used as the primary full-reference metrics. These metrics remain standard in dehazing and allow direct comparison with earlier and recent literature. To characterize efficiency, we report parameter count and FLOPs at the unified 256 × 256 setting as the primary complexity metrics. Hardware-dependent latency, FPS, memory, and high-resolution entries are reported as secondary deployment references under the same hardware protocol.

## 5. Results

The results are organized around four main evidence sources: paired-benchmark comparison, matched complexity analysis, module-level ablation, and kernel-size sensitivity evaluation. The quantitative claims are therefore based on paired benchmark performance, matched complexity analysis, module-level validation, and kernel-size sensitivity evaluation.

### 5.1. Quantitative Comparison on Paired Benchmarks

Table 1 reports the main quantitative comparison on SOTS-Indoor, SOTS-Outdoor, Dense-Haze, and NH-Haze. ADPCNet achieves 40.89 dB/0.997 on SOTS-Indoor and 37.80 dB/0.996 on SOTS-Outdoor while using 7.25 M parameters and 33.62 G FLOPs. Our method achieves a favorable quality-efficiency trade-off under the unified protocol. CasDyF-Net reports stronger RESIDE SOTS PSNR, and DehazeDDPM reports higher PSNR on Dense-Haze and NH-Haze; ADPCNet’s advantage is its compact complexity profile, higher SSIM on the two real paired datasets, and balanced coverage across the four paired benchmarks.

The SOTS-Outdoor result remains important because outdoor haze exhibits larger spatial variation and stronger semantic complexity than indoor synthetic haze. ADPCNet obtains 37.80 dB/0.996 on this benchmark, achieving competitive performance with OKNet while using fewer parameters and FLOPs. Compared with DehazeFormer-L, ADPCNet improves SOTS-Indoor PSNR by 0.84 dB while avoiding the substantially larger FLOP budget of transformer-style global mixing. The real paired results in Table 1 further complement the synthetic benchmark evaluation. On Dense-Haze and NH-Haze, ADPCNet achieves 18.05 dB/0.679 and 21.66 dB/0.815, respectively. Compared with OKNet, PMNet, DeHamer, and MSBDN, ADPCNet improves both PSNR and SSIM on the two real paired datasets, indicating that the proposed operator stack remains effective under real haze degradation. DehazeDDPM reports higher PSNR on Dense-Haze and NH-Haze, whereas ADPCNet achieves higher SSIM with a much smaller parameter budget. Therefore, within the unified Table 1 protocol, ADPCNet demonstrates a favorable quality-efficiency trade-off as a compact paired-supervised dehazing model.

ADPCNet recovers clearer visibility and more stable local structures under non-homogeneous haze while reducing color inconsistency and patchy amplification in challenging regions.

The upper examples show dehazing results under different haze densities, lighting conditions, and scene structures, while the lower examples illustrate typical difficult cases, including extremely dense haze, nearly invisible edges, local color shifts, and non-uniform illumination.

**Figure 4:**
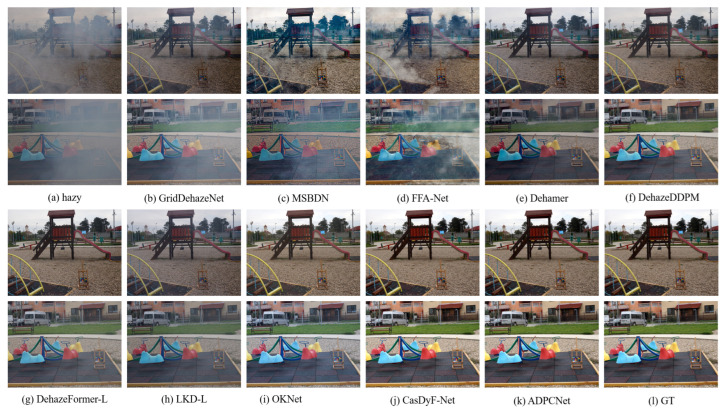
Representative real-world visual comparisons on Dense-Haze. (**a**): Hazy inputs. (**b**): GridDehazeNet. (**c**): MSBDN. (**d**): FFA-Net. (**e**): DeHamer. (**f**): DehazeDDPM. (**g**): DehazeFormer-L. (**h**): LKD-L. (**i**): OKNet. (**j**): CasDyF-Net. (**k**): ADPCNet. (**l**): Ground truth (GT).

**Figure 5:**
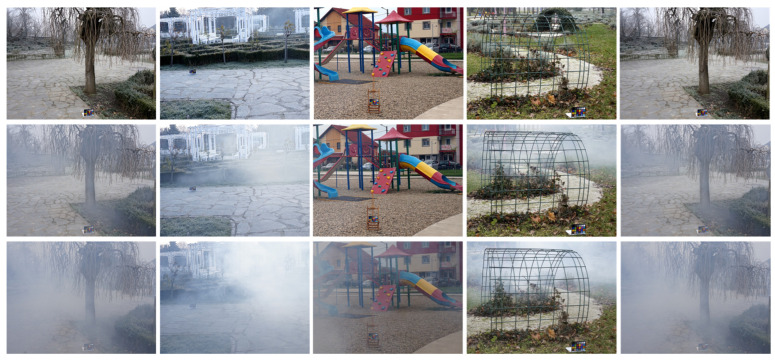
Additional qualitative results under diverse atmospheric conditions and representative challenging cases.

To further evaluate the robustness of ADPCNet beyond the representative visual examples, Figure 4 and Figure 5 provide additional qualitative comparisons under more diverse atmospheric conditions. The upper examples show that ADPCNet can maintain effective visibility restoration under different haze densities, lighting levels, and scene structures. These results complement the quantitative evaluation on SOTS, Dense-Haze, and NH-Haze by providing visual evidence under a broader range of degradation appearances.

The lower examples in Figure 5 further illustrate the applicability boundary of the proposed method. In extremely dense haze or regions where weak edges are nearly invisible, the restored images may still contain residual haze, blurred boundaries, or local color shifts. These cases indicate that ADPCNet improves real-scene visibility and structural consistency, but its restoration quality remains constrained by the amount of recoverable structural and textural evidence in the input.

### 5.2. Complexity Analysis

Table 2 reports the matched complexity anchor used in this paper. Under the unified 256 × 256 protocol, ADPCNet uses 7.25 M parameters and 33.62 G FLOPs, compared with 14.42 M parameters and 41.86 G FLOPs for OKNet. This corresponds to reductions of 49.7% in parameters and 19.7% in FLOPs relative to OKNet, while retaining competitive paired-benchmark performance. Although CasDyF-Net reports stronger RESIDE SOTS PSNR and DehazeDDPM reports higher PSNR on the real paired datasets, ADPCNet maintains a more compact complexity profile and achieves higher SSIM on the two real paired benchmarks. These results indicate that the proposed model provides a favorable quality-efficiency trade-off under a compact complexity profile.

**Figure 6:**
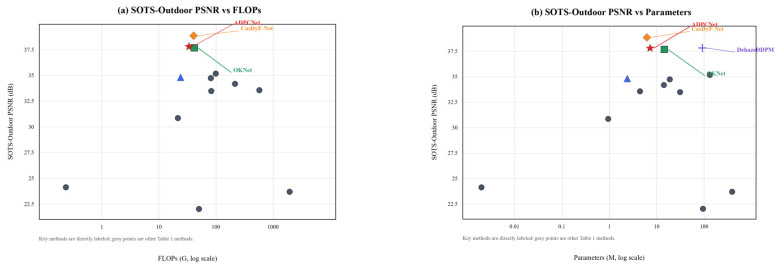
Matched complexity trade-off on SOTS-Outdoor using the Table 1 methods. (**a**): PSNR versus FLOPs. (**b**): PSNR versus parameters.

Figure 6 summarizes the matched parameter/FLOP comparison. Taken together, the two panels indicate that ADPCNet remains competitive in the matched complexity range, especially when paired-benchmark coverage on both synthetic and real-world datasets is considered together with parameter and FLOP cost.

### 5.3. Ablation and Sensitivity Study

Table 3 quantifies the contribution of each module on SOTS-Outdoor. Starting from the baseline network, CASM alone raises PSNR from 29.53 dB to 33.60 dB. DSM alone reaches 33.32 dB with slightly higher SSIM than the CASM-only model. FGM alone improves the baseline to 31.68 dB. These single-module results establish the numerical contribution of each component before multi-module fusion. The gains are not explained by parameter growth alone: CASM introduces a modest parameter increase but contributes the largest single-module gain, while DSM and FGM add smaller yet complementary improvements.

The module combinations further increase accuracy, which shows that the branches are complementary. Combining CASM and DSM increases PSNR to 35.98 dB, which is 6.45 dB higher than the baseline. Adding FGM to the CASM+DSM configuration yields 36.08 dB. The full model reaches 36.50 dB/0.9938 with 7.25 M parameters and the unified 33.62 G full-inference FLOPs. Compared with the baseline, the full configuration improves PSNR by 6.97 dB and SSIM by 0.0702 while increasing the parameter count by 1.75 M. The last step from row 8 to row 9 adds 0.42 dB and 0.0020 SSIM with only 0.03 M additional parameters, indicating that the final reconstruction stage benefits from aligned and fused branch features.

Table 4 examines the influence of the peripheral support size $k_{p}$ and the central independent kernel size $k_{c}$. Reducing $k_{p}$ from 63 to 31 lowers the computational cost but weakens PSNR and SSIM, indicating that insufficient peripheral support limits long-range haze-context modeling. Increasing $k_{p}$ to 95 brings only marginal improvement while increasing parameters and FLOPs. For the central region, $k_{c}=3$ reduces local modeling flexibility, whereas $k_{c}=11$ increases complexity without clear performance gain over $k_{c}=7$. Therefore, $k_{p}=63$ and $k_{c}=7$ are adopted as the default setting, providing a balanced trade-off among context range, local detail preservation, and computational efficiency.

## 6. Discussion

### 6.1. Why Adaptive Peripheral Large-Kernel Modeling Works for Dehazing

The main design premise of ADPCNet is that dehazing requires wide-range context estimation and local structure preservation at the same time. Atmospheric veiling is spatially smooth, but scene radiance changes abruptly at depth discontinuities, thin structures, and reflectance edges. A useful operator therefore needs broad spatial support while still allowing location-dependent responses. CASM addresses this requirement by combining a central independent kernel with gated peripheral sharing, so the receptive field expands without forcing every location to use the same response pattern.

Table 3 shows that the receptive-field component contributes the largest single-module gain in the current architecture. This numerical trend indicates that context modeling carries a large share of the restoration burden. The effect is too large to be explained by parameter increase alone, because the added capacity of CASM is modest relative to the size of the gain. The gate term then modulates how much peripheral sharing is used at each location, which is especially relevant outdoors, where sky, distant regions, and foreground texture require different amounts of contextual aggregation. The kernel-size sensitivity results in Table 4 further support this interpretation: insufficient peripheral support weakens context modeling, whereas excessively large support increases complexity with only marginal accuracy gain.

### 6.2. Role of Deformable Sampling and Frequency Guidance

DSM and FGM adjust the same backbone along two different axes. DSM changes where the large-kernel response is sampled, which matters most near depth boundaries and thin objects. Its ablation row slightly exceeds the CASM-only row in SSIM, which is consistent with better boundary consistency. FGM acts on the frequency response after contextual estimation, so its effect is most visible in faint edges, repetitive patterns, and local contrast transitions. Its standalone gain is smaller than the gains of CASM and DSM, but it remains useful after those two modules are combined, which shows that detail attenuation is not solved automatically by broader context or better sampling.

The full model performs best because these operations provide complementary internal representations for different stages of the restoration problem: wide-range visibility estimation, geometry-aware support adjustment, frequency-sensitive detail compensation, and adaptive evidence fusion. The ablation results show that the final DMF/detail stage adds only a small parameter increment, yet still improves the restoration result after the context, structure, and spectral cues have been aligned. Within the present architecture, the ablation results therefore support a staged and cooperative interpretation of ADPCNet: CASM contributes broad haze-context evidence, DSM adjusts its sampling support according to residual structure, FGM provides recoverable detail-compensation evidence, and DMF determines the spatial reliability of these cues before reconstruction.

### 6.3. Comparison with Recent Strong Baselines

The comparison places ADPCNet alongside current strong baselines instead of only historical CNN methods. However, recent dehazing methods are often evaluated under different protocols, including paired full-reference benchmarks, challenge-style NH-HAZE variants, unpaired real-world dehazing settings, and no-reference image-quality assessment. Therefore, the comparative analysis in this paper separates matched paired-benchmark evidence from broader protocol-aware discussion.

Within the matched paired setting, ADPCNet shows a favorable quality-efficiency trade-off. Relative to DeHamer and DehazeFormer-L, ADPCNet provides a convolution-centered alternative that avoids the substantially larger computational budget of heavy global-mixing architectures. Relative to OKNet and LKD-L, it introduces dehazing-specific adaptivity through deformable sampling and frequency-guided modulation. Relative to CasDyF-Net and DehazeDDPM, ADPCNet does not dominate every metric: CasDyF-Net reports stronger RESIDE SOTS PSNR, and DehazeDDPM reports higher PSNR on Dense-Haze and NH-Haze. Nevertheless, ADPCNet maintains a compact complexity profile while preserving balanced evaluation coverage across both synthetic and real paired datasets.

This positioning also clarifies the novelty of ADPCNet. Its main contribution lies in organizing large-context modeling, geometry-aware sampling correction, frequency-sensitive detail compensation, and dynamic branch fusion into a compact paired-supervised restoration framework. In this sense, ADPCNet provides a dehazing-oriented operator stack that balances visibility estimation, structural alignment, and detail recovery under a restrained computational budget.

### 6.4. Limitations

Despite its strong overall performance, ADPCNet still faces several technical limitations. First, like most supervised single-image dehazing models, ADPCNet may inherit a domain gap between synthetic training haze and real atmospheric scattering because its main training protocol relies on synthetic paired data. Consequently, the real paired benchmarks generally produce lower full-reference scores than the synthetic SOTS benchmarks, reflecting the greater difficulty of real haze degradation. Nevertheless, the results on Dense-Haze and NH-Haze show that ADPCNet remains competitive among directly comparable paired methods, particularly in terms of structural similarity and quality-efficiency balance. Dense-Haze and NH-Haze provide useful real-haze stress tests, but they cannot cover all possible atmospheric conditions; extremely unusual haze color, illumination level, fog density, or mixed-weather composition may still challenge the model. Second, DSM improves local adaptivity but cannot fully resolve all ambiguities in highly cluttered scenes with overlapping depth layers. Third, FGM enhances detail-sensitive responses, yet extremely weak textures that are almost completely submerged by dense haze remain difficult to recover without stronger priors or higher-resolution context. As in many previous restoration studies, under near-complete opacity, neither DSM nor FGM can recover contours or fine textures that are no longer observable in the input, because the necessary visual evidence is fundamentally insufficient.

High-resolution deployment is another practical limitation. Although ADPCNet is more efficient than several recent baselines, inference cost still scales with image area. Very large scenes may therefore benefit from tiled inference, cross-scale refinement, or memory-aware scheduling. These limitations define the next engineering targets for extending the model to harder deployment settings.

### 6.5. Implications for Broader Image Restoration

Although ADPCNet is developed for image dehazing, its design principles are relevant to a broader class of restoration problems. Many low-level vision tasks require the same combination of long-range contextual estimation, geometry-aware local aggregation, and high-frequency detail recovery. Denoising, deraining, low-light enhancement, and adverse-weather restoration all share this structure to varying degrees. In that sense, ADPCNet provides a concrete operator design for restoration settings in which broad context and local detail must be preserved simultaneously.

### 6.6. Future Work

Future work can extend ADPCNet along three practical directions. First, high-resolution deployment can be strengthened through native-resolution training and tiled inference refinement. Second, domain robustness can be improved by combining the present supervised design with lightweight real-world adaptation or no-reference regularization. Third, the adaptive sharing mechanism can be generalized from kernel weights to stage-wise routing so that the network adjusts both receptive field and compute allocation according to haze density. Additional future validation will include wider lighting levels, haze concentrations, fog types, and mixed-weather conditions to better quantify robustness beyond the current paired datasets.

## 7. Conclusions

This paper presented ADPCNet, a compact dehazing-oriented operator stack that separates three coupled subproblems of haze removal: broad context estimation, structure-aligned aggregation, and detail compensation. Instead of relying on dense large kernels or heavy full-attention backbones, ADPCNet organizes peripheral large-kernel context modeling, deformable sampling, frequency-guided modulation, and dynamic multi-branch fusion into a unified encoder-decoder framework. This design allows the network to model large-scale haze distribution, adjust sampling support around residual structures, and enhance recoverable high-frequency details within a restrained computational budget.

Under the matched paired evaluation protocol, ADPCNet uses 7.25 M parameters and 33.62 G FLOPs while achieving 40.89 dB/0.997 on SOTS-Indoor, 37.80 dB/0.996 on SOTS-Outdoor, 18.05 dB/0.679 on Dense-Haze, and 21.66 dB/0.815 on NH-Haze. In the ablation setting, the full configuration reaches 36.50 dB and 0.9938 with the same unified 33.62 G full-inference FLOP accounting. These results indicate that ADPCNet provides a favorable quality-efficiency trade-off as a compact paired-supervised dehazing model.

## Figures and Tables

**Table 1:** Quantitative comparison of representative dehazing methods on SOTS-Indoor, SOTS-Outdoor, Dense-Haze, and NH-Haze. Dataset columns report PSNR/SSIM. “--” denotes that the corresponding value was not reported in the original comparison source. Parameters and FLOPs follow the main ADPCNet comparison setting whenever the published papers report them. The compared methods have been introduced in Section 2.2, Section 2.3 and Section 2.4 according to their architectural categories.

Method	SOTS-In	SOTS-Out	Dense	NH	Params	FLOPs
SlaK-B	19.18/0.803	22.02/0.895	11.62/0.251	12.09/0.350	95.25	50.32
RepLKNet-XL	20.29/0.823	23.69/0.902	12.46/0.302	12.63/0.375	392.56	1958.16
AOD-Net	20.51/0.816	24.14/0.919	13.14/0.418	12.92/0.353	0.002	0.236
GridDehazeNet	32.14/0.983	30.86/0.982	13.31/0.365	13.80/0.543	0.95	21.49
MSBDN	33.77/0.984	33.48/0.982	15.37/0.493	19.23/0.713	31.35	83.08
FFA-Net	36.39/0.989	33.57/0.984	14.39/0.453	19.87/0.696	4.45	575.62
DeHamer	36.34/0.988	35.18/0.986	16.65/0.568	20.65/0.682	132.45	99.25
DehazeDDPM	38.02/0.988	37.82/0.986	19.04/0.592	22.28/0.731	92.62	--
MAXIM-S2	38.11/0.991	34.19/0.985	--	--	14.15	216.15
PMNet	38.41/0.990	34.74/0.985	16.79/0.515	20.42/0.733	18.90	81.13
DehazeFormer-L	40.05/0.996	--	--	--	25.44	560.36
LKD-L	39.44/0.994	34.82/0.983	--	--	2.38	23.93
OKNet	40.79/0.996	37.68/0.995	16.92/0.642	20.48/0.805	14.42	41.86
CasDyF-Net	43.21/0.997	38.86/0.995	--	--	6.23	40.55
ADPCNet	40.89/0.997	37.80/0.996	18.05/0.679	21.66/0.815	7.25	33.62

**Table 2:** Complexity-anchor table under the paper-reported 256 × 256 setting and the NVIDIA GeForce RTX 4090 deployment protocol. Parameter and FLOP columns are used as the primary cross-method complexity evidence, while latency, FPS, VRAM, and high-resolution entries provide secondary deployment references under the same hardware protocol.

Method	Params (M)	FLOPs @256 (G)	RTX4090 Lat. @256 (ms)	RTX4090 FPS @256	RTX4090 VRAM @256 (GB)	FLOPs @1024 (G)	Lat. @1024 (ms)	FPS @1024	VRAM @1024 (GB)
MSBDN	31.35	83.08	16.62	60.18	1.37	1329.28	265.86	3.76	7.26
DeHamer	132.45	99.25	19.85	50.38	3.23	1588.00	317.60	3.15	15.05
DehazeFormer-L	25.44	560.36	112.07	8.92	1.92	8965.76	1793.15	0.56	12.06
PMNet	18.90	81.13	16.23	61.63	1.15	1298.08	259.62	3.85	6.30
OKNet	14.42	41.86	8.37	119.45	0.95	669.76	133.95	7.47	5.06
LKD-L	2.38	23.93	4.79	208.94	0.66	382.88	76.58	13.06	3.60
CasDyF-Net	6.23	40.55	8.11	123.30	0.80	648.80	129.76	7.71	4.41
ADPCNet	7.25	33.62	6.72	148.72	0.79	537.92	107.58	9.30	4.28

**Table 3:** Ablation studies of each structural configuration on SOTS-Outdoor after 30-epoch training on RESIDE-Outdoor. CASM, DSM, FGM, and DMF + Detail denote the corresponding structural components, and the final row corresponds to the full ADPCNet configuration.

No.	Baseline	CASM	DSM	FGM	DMF + Detail	PSNR	SSIM	Params (M)	FLOPs (G)
1	Y	-	-	-	-	29.53	0.9236	5.50	25.69
2	Y	Y	-	-	-	33.60	0.9868	6.08	26.84
3	Y	-	Y	-	-	33.32	0.9885	5.82	27.15
4	Y	-	-	Y	-	31.68	0.9837	5.80	27.10
5	Y	Y	Y	-	-	35.98	0.9915	6.92	28.75
6	Y	Y	-	Y	-	34.53	0.9895	6.56	29.05
7	Y	-	Y	Y	-	34.35	0.9890	6.42	29.00
8	Y	Y	Y	Y	-	36.08	0.9918	7.22	30.05
9	Y	Y	Y	Y	Y	36.50	0.9938	7.25	33.62

**Table 4:** Sensitivity analysis of peripheral support size $k_{p}$ and central independent kernel size $k_{c}\;$ on SOTS-Outdoor.

$k_{p}$	$k_{c}$	Params (M)	FLOPs (G)	PSNR	SSIM
31	7	6.80	31.80	36.05	0.9929
63	3	7.15	32.50	36.20	0.9932
63	7	7.25	33.62	36.50	0.9938
63	11	7.40	34.40	36.48	0.9937
95	7	8.10	36.90	36.55	0.9939

## Data Availability

The benchmark datasets used in this study are publicly available from their official sources: RESIDE/SOTS (https://sites.google.com/view/reside-dehaze-datasets/reside-standard), Dense-Haze (http://www.vision.ee.ethz.ch/ntire19/dense-haze/), and NH-Haze (https://data.vision.ee.ethz.ch/cvl/ntire20/nh-haze/). No new dataset was generated in this study. The ADPCNet code, trained weights, inference scripts, and evaluation scripts have been organized for public release and will be made available in a public repository upon acceptance. Before public release, these materials are available from the corresponding author upon reasonable request.

## Abbreviations

The following abbreviations are used in this manuscript:

ADPCNet	Adaptive Deformable Peripheral Convolution Network
CASM	Conditional Adaptive Sharing Module
DSM	Deformable Sampling Module
FGM	Frequency-Guided Modulation
DMF	Dynamic Multi-branch Fusion
PSNR	Peak Signal-to-Noise Ratio
SSIM	Structural Similarity Index Measure
FLOPs	Floating Point Operations
FPS	Frames Per Second
FFT	Fast Fourier Transform
IFFT	Inverse Fast Fourier Transform
